# Substrates of the ASB2α E3 ubiquitin ligase in dendritic cells

**DOI:** 10.1038/srep16269

**Published:** 2015-11-05

**Authors:** Camille A. Spinner, Sandrine Uttenweiler-Joseph, Arnaud Metais, Alexandre Stella, Odile Burlet-Schiltz, Christel Moog-Lutz, Isabelle Lamsoul, Pierre G. Lutz

**Affiliations:** 1 CNRS; IPBS (Institut de Pharmacologie et de Biologie Structurale); 205 route de Narbonne BP 64182, F-31077 Toulouse, France; 2 Université de Toulouse, UPS, IPBS, F-31077 Toulouse, France

## Abstract

Conventional dendritic cells (cDCs) comprise distinct populations with specialized immune functions that are mediators of innate and adaptive immune responses. Transcriptomic and proteomic approaches have been used so far to identify transcripts and proteins that are differentially expressed in these subsets to understand the respective functions of cDCs subsets. Here, we showed that the Cullin 5-RING E3 ubiquitin ligase (E3) ASB2α, by driving degradation of filamin A (FLNa) and filamin B (FLNb), is responsible for the difference in FLNa and FLNb abundance in the different spleen cDC subsets. Importantly, the ability of these cDC subsets to migrate correlates with the level of FLNa. Furthermore, our results strongly point to CD4 positive and double negative cDCs as distinct populations. Finally, we develop quantitative global proteomic approaches to identify ASB2α substrates in DCs using *ASB2* conditional knockout mice. As component of the ubiquitin-proteasome system (UPS) are amenable to pharmacological manipulation, these approaches aimed to the identification of E3 substrates in physiological relevant settings could potentially lead to novel targets for therapeutic strategies.

Present in blood, in mucosae and in lymphoid organs, DCs play the double role of sentinels and conductors of the immune system. Indeed, immature DCs recognize and capture antigens from infectious agents, cancer cells or self-antigens. Once activated by antigen uptake and exposure to inflammatory stimuli, DCs initiate a maturation program that culminates with the activation of T cells to set up a specific immune response. Although DCs are heterogeneous, most of them are from hematopoietic lineage deriving from hematopoietic stem and progenitor cells. Indeed, Macrophage and DC precursors (MDP) differentiate into common DC precursors (CDP) that give rise to plasmacytoid and pre-DCs in the bone marrow^1^,^2^,^3^. Pre-DCs migrate through the blood to seed lymphoid and non-lymphoid organs where they differentiate into cDCs^4^,^5^. The lymphoid tissue-resident cDCs can be divided into three major subpopulations based on the expression of CD8α and CD4, the CD4^+^CD8α^−^, CD4^−^CD8α^+^ and CD4^−^CD8α^−^ DC subsets. However, their precise function is not fully understood. In this context, identification of regulators of the different DC subsets should contribute to our understanding of their respective functions. Differentiation of pre-DC in these distinct subsets is controlled by micro-environmental signals. Splenic CD4^+^ cDCs that are localized in the marginal zone are involved in the capture of blood-borne antigens^6^,^7^. Splenic CD8α^+^ cDCs have a higher ability to cross-present exogenous antigen on MHC class I molecules and prime CD8^+^ T cells whereas CD4^+^ and CD4^−^CD8α^−^ cDCs are more efficient in priming CD4^+^ T cells through MHC class II molecules^6^,^8^. Our recent work points to the E3 ASB2α and its substrates, the actin-binding protein filamins (FLNs), as new regulators of DCs^9^.

Protein ubiquitylation is a reversible post-translational modification that directs proteins to a myriad of fates such as targeting proteins to degradation by the proteasome, internalization, lysosomal targeting, modulating protein interactions or regulation of signaling pathways and transcription. Ubiquitylation occurs through the sequential action of an E1 ubiquitin-activating enzyme, an E2 ubiquitin-conjugating enzyme and an E3. In this pathway, E3s provide platforms for binding an E2 ubiquitin-conjugating enzyme and specific substrates, thereby coordinating the ubiquitylation of the selected protein. Not surprisingly, ubiquitylation has been linked to all cellular processes and defects in ubiquitylation pathways can contribute to disease pathogenesis. These make E3s attractive therapeutic targets^10^,^11^. Ubiquitin-mediated protein degradation is the major controlled proteolytic pathway in Eukaryotes. Its specificity derives from the many hundreds of E3s that recognize specific substrates to be degraded. Identification of the substrate(s) of E3s targeted to proteasomal degradation in a given cell type is a crucial issue to understand E3 functions and mechanisms of action. Purification of interacting proteins is the classical way to identify substrates of E3s. Although this had successfully lead to the identification of a number of substrates of monomeric E3s, identification of substrates of multimeric E3s is very challenging because of the weak affinity of substrates for their requisite specificity subunit and because of the labile nature of the substrate complexed with the specificity subunit^12^. In this context, we have previously developed a strategy for the identification of substrates of E3s that are targeted to proteasome degradation using cell lines induced to express an active or a defective ubiquitin ligase mutant of an E3^13^.

We previously showed that ASB2α is the specificity subunit of a Cullin 5 RING E3 suggesting that ASB2α exerts its effects through the targeting of specific substrates for degradation by the proteasome^14^. Indeed, ASB2α E3 activity drives proteasome-mediated degradation of FLNs and can regulate integrin-dependent functions such as cell spreading, cell adhesion and initiation of cell migration of several cell lines^13^,^15^,^16^,^17^,^18^. These findings have now been confirmed by others^19^,^20^. We recently demonstrated that ASB2α transcripts are expressed in mouse primary immature DCs generated *ex vivo*, the so-called bone marrow-derived DCs (BMDCs), and down-regulated following BMDC activation^9^. Using BMDCs from *ASB2* conditional knockout mice, we further demonstrated that ASB2α triggers degradation of FLNa and FLNb in BMDCs and that ASB2α regulates BMDC migration by promoting extracellular matrix proteolysis^9^.

Here we showed that FLNa and FLNb are the main substrates of ASB2α in mouse splenic cDCs and that ASB2α, by driving degradation of FLNa and FLNb, is responsible for the difference in levels of FLNa and FLNb in the different DC subsets. Moreover, the ability of cDC subsets to migrate correlates with the level of FLNa. Furthermore, we proposed and validated a label free quantitative proteomic approach to identify E3 substrates to be degraded in physiological relevant settings.

## Results

### ASB2α is expressed in mouse conventional DCs

**Figure 1:**
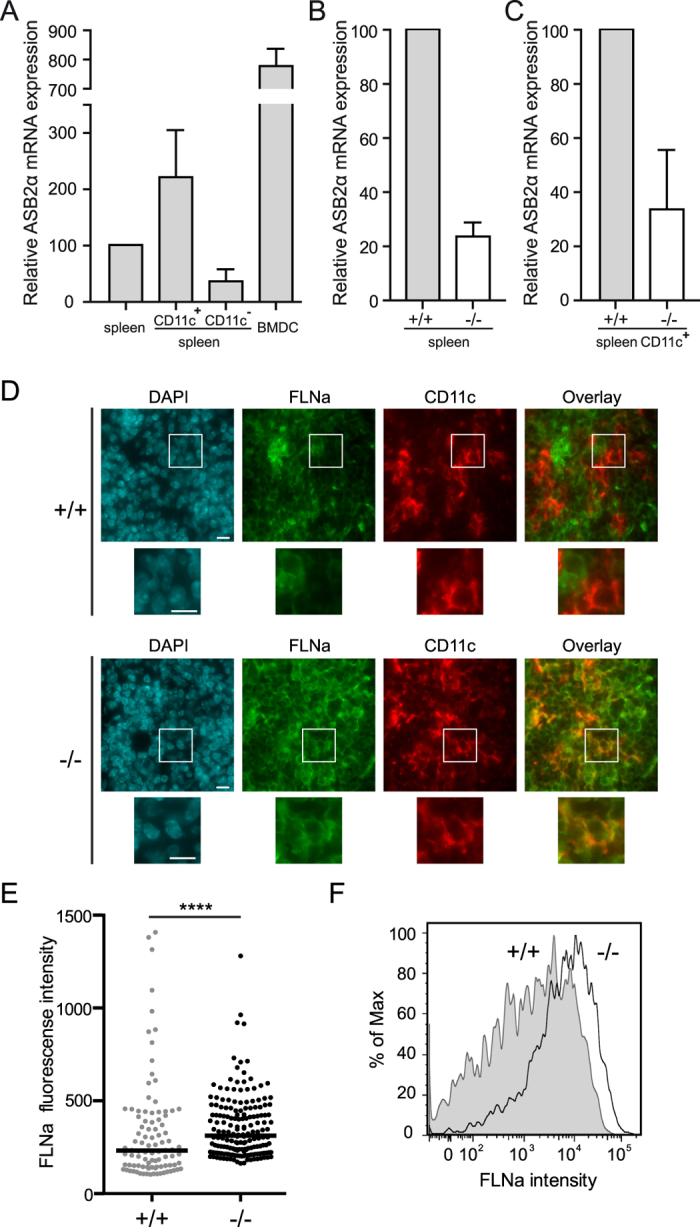
ASB2α triggers FLNa degradation in mouse spleen conventional DCs. (**A–C**) Relative expression of ASB2α mRNA assessed by RT-qPCR in spleen, FACS-purified CD11c positive and negative spleen cells and BMDCs of control mice (**A**) and relative expression of ASB2α mRNA in splenocytes (**B**) or FACS-purified CD11c positive spleen cells of Mx1-Cre (+/+) and Mx1-Cre;ASB2fl/fl (−/−) mice that have received poly(I·C) (indicated as +/+ and −/−, respectively). Levels were normalized to Arbp. The data show means and SEM of three independent experiments (sample size: +/+ = 15; −/− = 14 except for BMDCs, sample size = 8). (**D**) Frozen sections of the spleen of Mx1-Cre (+/+) and Mx1-Cre;ASB2^fl/fl^ (−/−) mice that have received poly(I·C) were examined for FLNa, CD11c and DAPI staining. Magnified views are also showed. Scale bars represent 10 μm. One representative experiment is presented. (**E**) Quantitation of FLNa expression assessed by immunofluorescence in CD11c positive spleen cells of Mx1-Cre (+/+) and Mx1-Cre;ASB2^fl/fl^ (−/−) mice that have received poly(I·C). Cells were centrifuged onto glass slides, fixed and stained for FLNa. Dot plots show the overall distribution of relative FLNa fluorescence intensities, and lines shows the median values. The p-value was calculated using the Mann-Whitney t-test. ****p< 0.0001. (**F**) Expression of FLNa was assessed by intracellular flow cytometry coupled to extracellular flow cytometry in CD11c high spleen cells. After fixation and permeabilization, cells were stained with anti-FLNa and brilliant violet 421-conjugated anti-rabbit antibodies. Gray and white areas show representative FLNa staining profiles in CD11c high (CD11c^hi^) cells of Mx1-Cre (+/+) and Mx1-Cre;ASB2^fl/fl^ (−/−) mice that have received poly(I·C), respectively. One representative experiment out of three is presented.

Because microarray studies indicated that *ASB2* mRNAs are expressed in mouse conventional DCs isolated from lymphoid or non-lymphoid tissues^21^,^22^ and because mRNAs of the alpha isoform of *ASB2* were expressed in mouse immature bone marrow- and spleen-derived DCs^9^, we evaluated the expression of ASB2α mRNAs in DCs purified from mouse spleens. As controls, we used *ASB2* knockout DCs isolated from Mx1-Cre;ASB2^fl/fl^ mice that were injected with synthetic double-stranded RNA poly(I·C) to activate the Mx1 promoter in hematopoietic stem progenitor cells^9^. Mx1-Cre mice that have received poly(I·C) were used as ASB2^+/+^ control mice. Six weeks after the last poly(I·C) injection, spleen cells were analyzed for ASB2α mRNA expression (Fig. 1A–C). DCs were sorted as CD11c^+^ cells by flow cytometry. Indeed, analysis of FACS-purified CD11c positive cells from Mx1-Cre and Mx1-Cre;ASB2^fl/fl^ mice that have received poly(I·C) revealed that ASB2α transcripts are expressed in CD11c^+^ cells (Fig. 1A,C). Expression of ASB2α transcripts segregated predominantly to the CD11c^+^ fraction of Mx1-Cre control mice and, as expected, expression of ASB2α was drastically decreased in CD11c^+^ cells sorted from spleen of Mx1-Cre;ASB2^fl/fl^ mice that have received poly(I·C) (Fig. 1A,C). These cells are hereafter referred to as ASB2α^−/−^ DCs. Similar results were obtained in FACS-purified CD11c^+^ cells isolated from mouse bone marrows (data not shown).

### FLNa is a substrate of ASB2α in mouse conventional DCs

**Figure 2:**
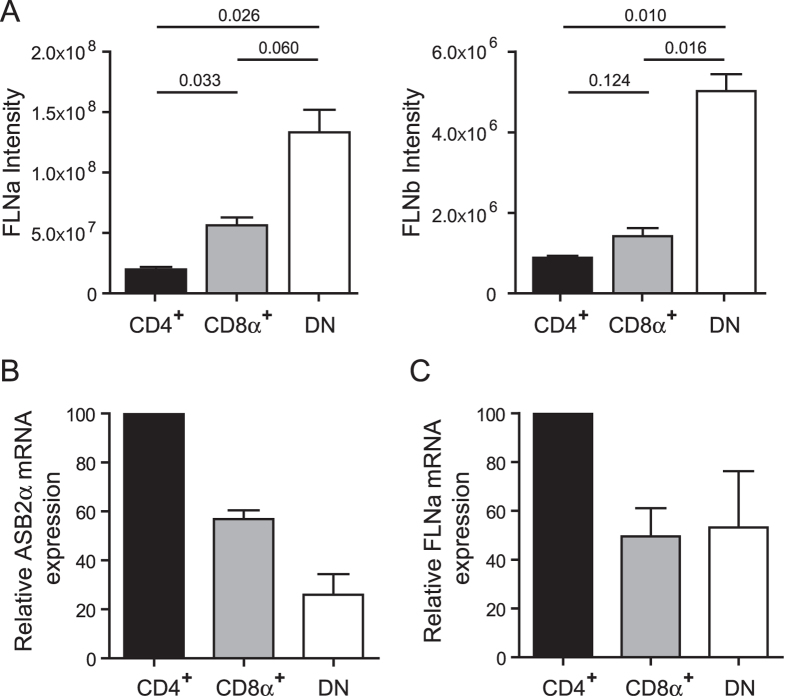
Expression of FLNa and ASB2α in mouse spleen DC subsets. (**A**) Intensities of FLNa (left panel) and of FLNb (right panel) calculated using MaxQuant quantitative metrics in CD11c^hi^CD4^+^CD8α^−^ (CD4^+^), CD11c^hi^CD4^−^CD8α^+^ (CD8α^+^) and CD11c^hi^CD4^−^CD8α^−^ (DN) extracts. p-values were calculated with the unpaired t-test with Welch’s correction. Data plotted are from Luber *et al.*^23^ with courtesy of Meredith O’Keeffe. (**B,C**) Relative expression of ASB2α (**B**) and FLNa (**C**) mRNA assessed by RT-qPCR in CD4^+^, CD8α^+^ and DN cDC subsets in spleen of ASB2^+/+^ mice. Levels were normalized to Arbp. The data show means and SEM of three independent experiments (sample size: +/+ = 16).

**Figure 3:**
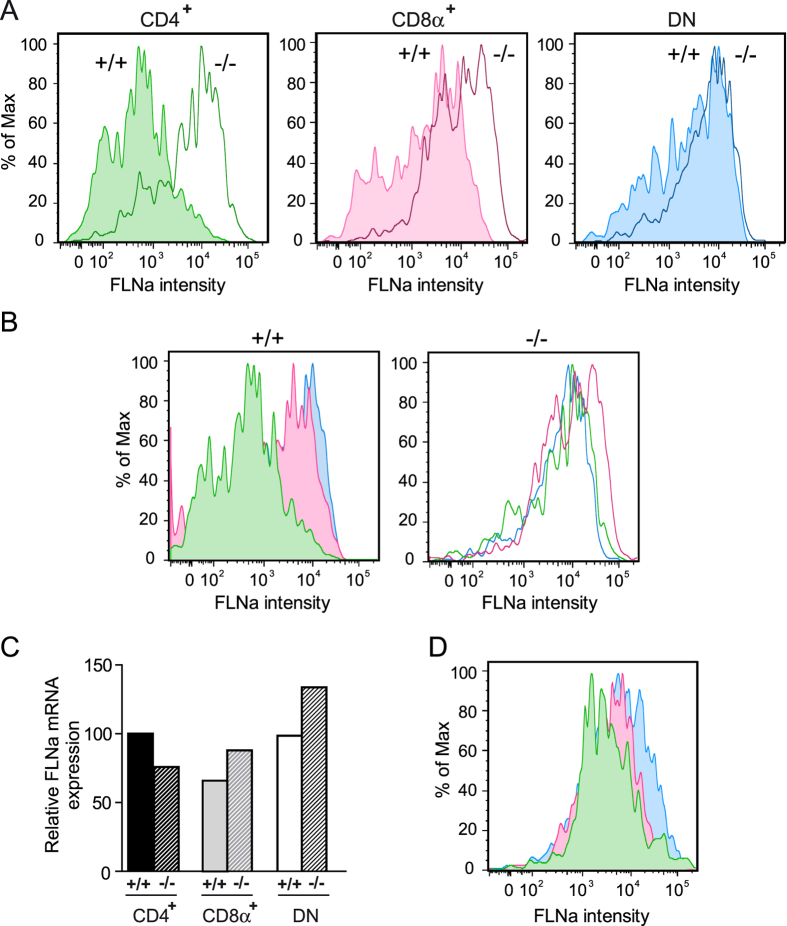
ASB2α mediates FLNa degradation in cDC subsets. (**A,B**) Expression of FLNa was assessed by intracellular flow cytometry coupled to extracellular flow cytometry in CD11c^hi^CD4^+^CD8α^−^ (CD4^+^; green), CD11c^hi^CD4^−^CD8α^+^ (CD8α^+^; pink) and CD11c^hi^CD4^−^CD8α^−^ (DN; blue) spleen cDC subsets from Mx1-Cre (+/+) and Mx1-Cre;ASB2^fl/fl^ (−/−) mice that have received poly(I·C). Filled histograms show expression of FLNa in cDC subsets of control mice whereas unfilled histograms show expression of FLNa in cDC subsets of Mx1-Cre;ASB2^fl/fl^ mice that have received poly(I·C) (sample size: +/+ = 3; −/− = 4). One representative experiment out of three is presented. (**C**) Relative expression of FLNa mRNA assessed by RT-qPCR in CD4^+^, CD8α^+^ and DN cDC subsets in spleen of Mx1-Cre (+ /+ ) and Mx1-Cre;ASB2^fl/fl^ (− /− ) mice that have received poly(I·C) (sample size: +/+ = 6; −/− = 6). (**D**) Expression of FLNa was assessed by intracellular flow cytometry coupled to extracellular flow cytometry in CD4^+^, CD8α^+^ and DN cDCs isolated from mesenteric lymph nodes of ASB2^+/+^ mice (sample size = 5). One representative experiment out of three is presented.

Immunohistochemistry analyses of spleens of ASB2α^+/+^ mice demonstrated the absence or the very low levels of the FLNa protein in CD11c^+^ cells (Fig. 1D). In contrast, expression of FLNa was higher in CD11c^+^ cells of Mx1-Cre;ASB2^fl/fl^ mice that have received poly(I·C) (Fig. 1D). To additionally confirm these data and to provide more quantitative information regarding the efficiency of ASB2α-mediated FLNa degradation in ASB2α^+/+^ spleen DCs, quantification of the expression of FLNa was performed in cytospun DCs by immunofluorescence microscopy. Figure 1E shows FLNa expression profiles in ASB2α^+/+^ and ASB2α^−/−^ CD11c^+^ cells. Knockout of ASB2α in DCs resulted in increased levels of FLNa compared to ASB2α^+/+^ DCs (Fig. 1E). This was further confirmed by intracellular flow cytometry coupled to extracellular flow cytometry (Fig. 1F). As shown in Fig. 1D-F, the levels of FLNa in spleen ASB2α^+/+^ CD11c^+^ cells were heterogeneous. Because cDCs can be divided according to the expression of the surface markers CD4 and CD8α, we investigated the status of FLNa in CD11c^+^CD4^+^, CD11c^+^CD8α^+^ and CD11c^+^CD4^−^CD8α^−^ (double negative, DN) spleen cDCs. In fact, MS-based proteomics combined with label-free quantitation algorithms aimed to determine the proteome of mouse spleen cDC subsets^23^ indicated that DN DCs expressed higher levels of both FLNa and FLNb than CD4^+^ and CD8α^+^ DCs (Fig. 2A). To investigate whether ASB2α may be responsible for the differential expression of FLNa and FLNb in cDCs populations, RT-qPCR were performed to measure the abundance of ASB2α and FLNa transcripts in the CD4^+^, CD8α^+^ and DN cDCs (Fig. 2B,C). A shown in Fig. 2A,B, (i) among cDCs, the CD4^+^ population that expressed the lowest levels of FLNa and FLNb expressed the highest levels of ASB2α mRNA, (ii) ASB2α mRNA expression was low in the DN cDC subset that expressed the highest levels of FLNa and FLNb, (iii) CD8α^+^ cDCs expressed intermediate levels of ASB2α mRNA, FLNa and FLNb. In agreement with these results, few peptides of ASB2α were exclusively detected in CD4^+^ and CD8α^+^ cDCs^23^. Furthermore, CD4^+^ cDCs expressed higher levels of FLNa transcripts than CD8α^+^ and DN cDCs (Fig. 2C) indicating that the reduced levels of FLNa protein in CD4^+^ cDCs is not due to reduced levels of FLNa transcripts in this subset. Altogether, these results strongly suggest that ASB2α-mediated FLNa and FLNb degradation is responsible for the differential abundance of FLNa and FLNb in cDCs populations. We therefore investigated FLNa expression in CD4^+^, CD8α^+^ and DN cDCs of Mx1-Cre;ASB2^fl/fl^ and Mx1-Cre mice that have received poly(I·C) by intracellular flow cytometry. Percentages of the different cDC subsets were similar in the spleen of Mx1-Cre;ASB2^fl/fl^ and control mice that have received poly(I·C) (data not shown). In agreement with the MS-based proteomic approach, intracellular flow cytometry analyses revealed that the level of FLNa was lower in CD4^+^ than in CD8α^+^ and DN cDCs of ASB2α^+/+^ mouse spleens (Fig. 3A,B). Furthermore, the levels of FLNa were similar in the different cDC subsets from ASB2α^−/−^ mouse spleens and in their counterparts from ASB2α^+/+^ mouse spleens (Fig. 3A,B). These results showed that loss of ASB2α resulted in increased levels of FLNa. We also observed that FLNa transcripts in the different spleen cDC populations are not affected by ASB2 knockout (Fig. 3C). Collectively, our results demonstrated that ASB2α is responsible for the differential abundance of FLNs in cDC populations. Interestingly, different levels of FLNa were also observed in CD4^+^, CD8α^+^ and DN DCs isolated from mesenteric lymph nodes, with the highest levels in DN and the lowest in CD4^+^ cDCs (Fig. 3D).

### Migratory properties of cDC subsets correlate with FLNa levels

**Figure 4:**
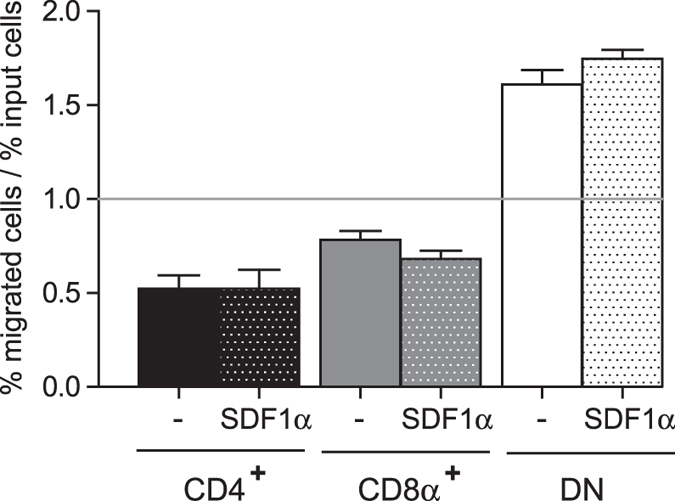
Migratory properties of spleen cDC subsets. CD11c^+^ spleen cells of ASB2^+/+^ mice were plated into transwell inserts and allowed to chemotax towards medium containing or not SDF1α, as indicated. After 2 hours at 37 °C, migrated cells were recovered from the wells and quantified by flow cytometry together with input cells. For each cDC subset, the ratio between the percentage of migrated cells and the percentage of input cells as well as SEM of three independent experiments are shown (sample size = 16). The line indicates the 1:1 ratio achieved where all cDC subsets migrate with equal efficiency.

Since FLNa is an important regulator of cell migration^24^, we next asked whether the differential expression of FLNa in cDC populations has an impact on their migratory properties. CD11c^+^ spleen cells were added to the top well of a transwell chamber in the presence or absence of SDF1α added to the bottom compartment. After 2 h, cells that had migrated to the lower chamber were recovered and the percentages of each DC subsets were quantitated by FACS along with input cells. Although the inclusion of SDF1α boosted migration of each DC subsets (2-fold increase, data not shown), the migration of DN cDCs was higher than the migration of CD8α^+^ cDCs and the migration of CD8α^+^ cDCs was higher than the migration of CD4^+^ cDCs in the absence or presence of SDF1α (Fig. 4). This suggests that the migratory properties of cDCs correlate with FLNa levels and that FLNa has a positive effect on the migration of spleen cDCs.

### Quantitative proteomic comparison of ASB2α^+/+^ and ASB2α^−/−^ DCs

**Figure 5:**
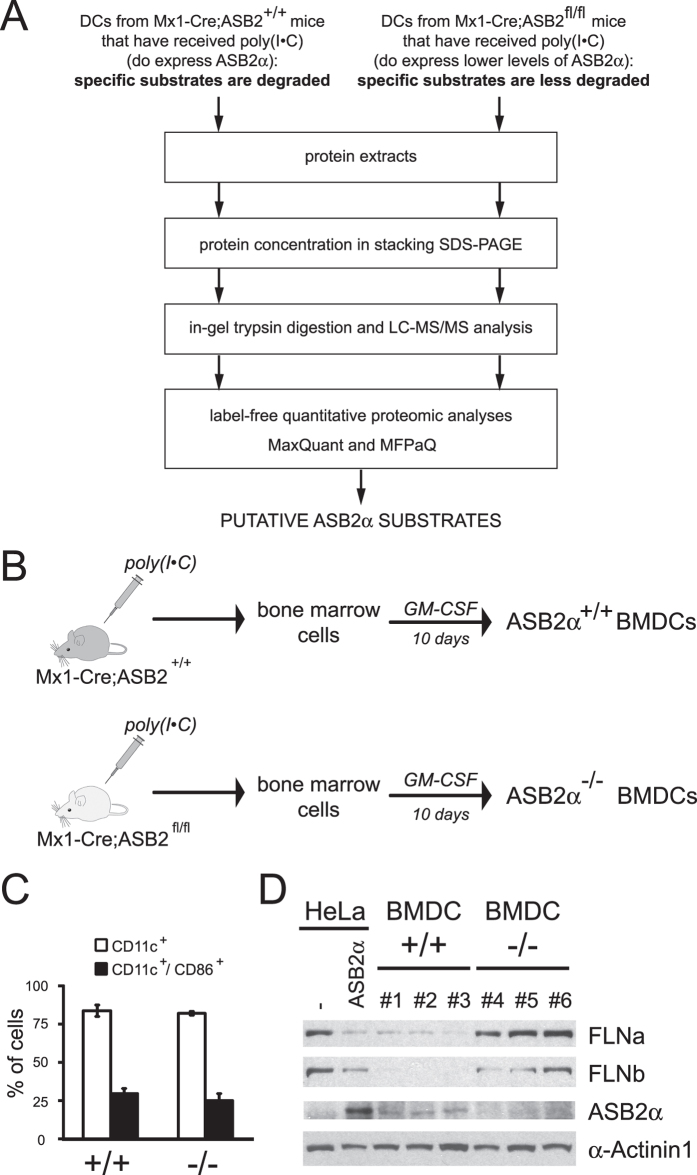
Experimental design for identification of ASB2α substrates in DCs. (**A**) Experimental design. (**B**) Experimental outline of ASB2 deletion and generation of GM-CSF BMDCs. (**C**) Expression of CD11c and CD86 at the cell surface of ASB2α^−/−^ and ASB2α^+/+^ BMDCs. (**D**) Expression of FLNa, FLNb, ASB2α, α-actinin 1 was analyzed by western blot using 10-μg aliquots of protein extracts of ASB2α^−/−^ and ASB2α^+/+^ BMDCs from three independent experiments and 3-μg aliquots of cell extracts of HeLa cells transfected with a mouse ASB2α expression vector (ASB2α) or mock-transfected (−). The drawing in panel B is from P.G.L.

**Figure 6:**
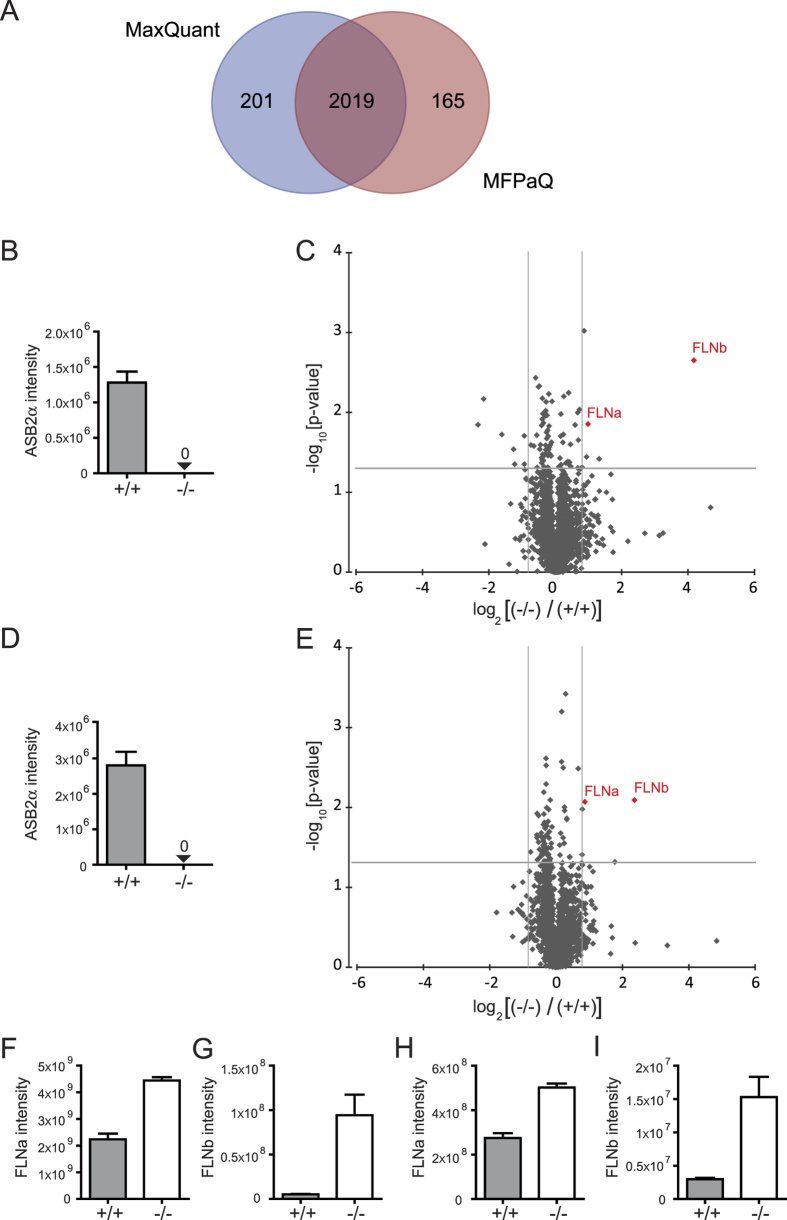
Quantitative protein expression differences between ASB2α^−/−^ and ASB2α^+/+^ BMDCs. (**A**) Venn diagram that shows the overlap between proteins quantified with the two quantitative software, MFPaQ and MaxQuant. (**B,D**) ASB2α intensities from MaxQuant (**B**) and MFPaQ (**D**) quantitative analyses for ASB2α^+/+^ and ASB2α^−/−^ BMDC extracts. 0 indicates that no peptide was attributed to the ASB2α protein. (**C,E**) Volcano plots from MaxQuant (**C**) and MFPaQ (**E**) quantitative analyses of protein expression differences between ASB2α^−/−^ and ASB2α^+/+^ BMDCs as a function of statistical significance. Pointed lines mark threshold limits (log_2_[ratios ASB2α^−/−^ *vs.* ASB2α^+/+^]>0.85 or < 0.85; t-test p-value < 0.05) for up- or downregulated proteins. Proteins highlighted in red were found to be variant using both software. (**F–I**) Intensities of FLNa (**F,H**) and of FLNb (**G,I**) calculated using MaxQuant (**F,G**) and MFPaQ (**H,I**) quantitative metrics for ASB2α^+/+^ and ASB2α^−/−^ BMDC extracts.

Whether other proteins are substrates of ASB2α in DCs is critical to understand ASB2α function(s) in DCs. We therefore identified differentially expressed proteins among ASB2α^+/+^ and ASB2α^−/−^ DCs using a MS-based proteomic approach. Indeed, substrates of ASB2α are expected to be degraded in ASB2α^+/+^ DCs but to accumulate in ASB2α^−/−^ DCs (Fig. 5A). We used as a model system GM-CSF BMDCs generated from bone marrow cells of Mx1-Cre and Mx1-Cre;ASB2^fl/fl^ mice that have received poly(I·C) (Fig. 5B). Three independent experiments were carried out, resulting in biological triplicates. Surface expressions of the DC marker CD11c and of the activation marker CD86 were similar in BMDCs obtained from each culture (Fig. 5C), showing that the loss of ASB2α has no impact on the generation and activation of DCs. As controls, we first evaluated the expression of ASB2α protein and its well-established substrates, FLNa and FLNb, by immunoblotting. As shown in Fig. 5D, ASB2α-expressing BMDCs expressed low levels of FLNa and FLNb. In contrast, ASB2α was not detected in ASB2α^−/−^ BMDCs while FLNa and FLNb were highly expressed. After protein reduction and alkylation, 50 μg of BMDC extracts from 3 biological replicates of ASB2α^+/+^ and ASB2α^−/−^ BMDCs were concentrated in 4.4% stacking polyacrylamide gels. After tryptic in-gel digestion, the resulting peptides were analyzed by nanoLC-MS/MS. MS data of the three independent experiments were combined and analyzed by two label-free quantitation algorithms, MaxQuant^25^ and MFPaQ^26^,^27^. Relative label-free quantitation with these two bioinformatics tools was highly reproducible between biological replicates since correlation between normalized protein intensities was between 0.95 and 0.98 (Figure S1). Moreover, a majority of the proteins quantified by MFPaQ (2184 proteins) or by MaxQuant (2220 proteins) were in common (2019 proteins) (Fig. 6A). No expression of the ASB2α protein was detected in BMDCs generated from bone marrow cells of Mx1-Cre;ASB2^fl/fl^ mice that have received poly(I·C) (Fig. 6B,D), in agreement with our previous data showing a drastic decreased of ASB2α mRNA levels in these cells^9^. Expression of most proteins was similar and only a very small number showed statistically significant differences between ASB2α^+/+^ and ASB2α^−/−^ BMDCs (Fig. 6C,E, Table S1). Differential proteins with over 1.8-fold changes associated to p-values <0.05 by both MaxQuant and MFPaQ approaches, were considered as potential candidates. Surprisingly, only two proteins that accumulated in ASB2α^−/−^ *vs* ASB2α^+/+^ BMDCs were highlighted by the two analyzes, FLNa and FLNb (Fig. 6C,E–I). This further stresses that FLNs are the most robust and highly validated substrates of ASB2α.

## Discussion

Here, we showed that transcripts of the ASB2α isoform are expressed in mouse splenic cDCs in agreement with previous microarray data^21^,^22^ and that the E3 ubiquitin ligase ASB2α, by driving degradation of FLNa and FLNb, is responsible for the difference in FLNa and FLNb abundance in the different DC subsets. Indeed, cDCs are heterogeneous and comprise distinct subsets that may have evolved to serve distinct functions. Therefore, identification of proteins that are differentially expressed in these subsets should contribute to our understanding of their respective functions. In fact, the CD4^+^ and DN cDC subsets are often gathered in a CD8α^−^CD11b^+^ DC population since they are more closely related to each other than to the CD8α^+^ cDC subset as shown by microarray^22^ and proteomic^23^ analyses. The finding that FLNa accumulates in spleen cDCs of *ASB2* inducible knockout mice indicated that FLNa is targeted for proteasomal degradation by ASB2α in these cells. Therefore, the level of ASB2α is likely responsible for the level of FLNs in the different spleen cDC subsets. Interestingly, differential expression of FLNa in the different cDC populations isolated from mesenteric lymph nodes was similarly observed. Our results are also in agreement with data of a global proteomic approach showing that the ratio of the levels of both FLNa and FLNb in DN *vs* CD4^+^ cDCs are among the highest ratios found in the whole proteome of these DC subsets (7.10 ± 1.75 and 5.90 ± 1.30, respectively)^23^. These are also consistent with the fact that ASB2α peptides were detected in CD4^+^ and CD8α^+^ but not in DN DCs^23^. Altogether, these results indicate that CD4^+^ and DN DCs are therefore likely to be distinct populations and that the CD8α^−^CD11b^+^ DC subset should be segregated into CD4^+^ and DN DC subsets. In fact, different transcription networks regulate DCs subsets^28^. Two signalling pathways activated by environment factors were recently showed to influence pre-cDC commitment between alternative DC subsets: (i) retinoic acid signalling^29^,^30^ and (ii) Notch signalling^31^,^32^,^33^. Importantly, these two pathways are known to activate *ASB2* expression in hematopoietic cells^34^,^35^,^36^. Whether these pathways are involved in the regulation of *ASB2* expression in DCs remains to be established. Differences in FLN abundance between cDC subsets may have impacts in DC functions. Our previous results^9^ together with those reported herein demonstrate a mechanism of regulation of FLN stability by ASB2α in DCs. Given the facts that ASB2α mediates FLN degradation in DCs and that FLNs are implicated in actin cytoskeleton organization, in cell shape and in cell motility, our data raise the possibility that FLN abundance may account for functional differences between DC subsets. Indeed, we showed that the migratory properties of CD4^+^, CD8α^+^ and DN cDCs correlate with FLNa levels and that FLNa has a positive effect on the migration of spleen cDCs. This is in agreement with our previous results showing a role of FLNs in initiation of cell migration^16^.

Although FLNs are now well-established substrates of ASB2α in physiological relevant settings, whether other proteins are targeted to proteasomal degradation by ASB2α is still an open question. In fact, identification of substrates of E3 ubiquitin ligases targeted for degradation is a tricky task, particularly for those of the Cullin RING ligase family. It is important to mention that most experiments described in the literature are carried out by overexpressing the ubiquitin ligase and/or its substrate candidate, often in non-physiological models. We therefore set up an approach aimed to the identification of substrates of E3 ubiquitin ligases to be degraded in physiological relevant settings. Indeed, we used a shotgun proteomic approach to identify proteins that accumulate in ASB2α-deficient DCs (because they are not or less degraded) but not in ASB2α-expressing DCs (because they are degraded). Because bioinformatics analysis of label-free quantitative proteomic data is still challenging and can produce false positive results, we hypothesized that the analysis via two different bioinformatics software may reduce the list of false positive protein candidates. Indeed, comparison of the results obtained using MaxQuant and MFPaQ tools showed that FLNa and FLNb are the only proteins that are more expressed in ASB2α^−/−^ DCs than in ASB2α^+/+^ DCs. The fact that FLNa and FLNb were the only ASB2α substrate candidates identified in our global proteomic approach strongly suggests a high selectivity of ASB2α for FLNa and FLNb, at least in hematopoietic cells. Furthermore, our results indicate that the combination of different bioinformatics tools may help to obtain more confident results, and to select candidate substrates of E3s targeted to proteasomal degradation for further validation. We previously provided a global quantitative proteomics strategy for the identification of E3 substrates to be degraded based on the comparison of the proteomes of cell lines expressing a wild-type E3 or an E3 defective mutant^13^. Here, we propose an up-dated and improved version of this approach that is applicable to primary cells or tissues and that does not rely on overexpression experiments. Indeed, our study demonstrates that label-free quantitation approaches to compare amounts of thousands of proteins in mouse primary cells of an E3 ubiquitin ligase knockout and a wild-type mouse can be used to identify substrates of the E3 that are targeted to degradation.

## Methods

### Mice, induction of *ASB2* hematopoietic knockout, DC isolation and generation

All mice^9^ were bred under specific pathogen-free conditions. The experiments on mice were handled according to the Centre National de la Recherche Scientifique ethical guidelines and approved by the Midi-Pyrénées Regional Ethical Committee. At an age between 6 and 8 weeks, mice of the desired genotype received three injections at 2-days intervals of 300 μg polyinosinic-polycytidylic acid [poly(I·C); Sigma Aldrich]. Mice were killed 6 weeks after the last injection. Spleen single cell suspensions were obtained with the gentleMACS™ Dissociator as recommended by the manufacturer (Miltenyi Biotec) and red blood cells were lysed in 150 mM NH_4_Cl, 10 mM KHCO_3_, 0.1 mM EDTA (pH 7.2). Mesenteric lymph nodes were digested in RPMI 1640 containing 0.2 mg/ml of collagenase D (Roche) and 0.1 mg/ml of DNase-I (Roche) for 20 min at 37 °C. Single cell suspensions were generated from digested tissues after filtration through a 70 μm-strainer. GM-CSF BMDCs were generated from mouse bone marrow cells as previously described^9^.

### Antibodies

Antidodies against surface markers were: CD11c APC and biotin (N418), CD4 FITC (RM4-5), CD8α PE (53-6.7) and CD86 PE (GL1) (all from Biolegend). Isotype-matched antibodies were used as controls. The monoclonal rabbit anti-FLNa from Genetex was used for immunohistochemistry. The rabbit anti-FLNa serum raised against bacterially expressed recombinant human FLNa d16–20 and absorbed against the homologous fragments of human FLNb and FLNc was used for flow cytometry^18^. The anti-human FLNa serum that cross-reacts with mouse FLNa was used for immunofluorescence experiments and immunobloting^15^. Anti-FLNb (N-16) and anti-α-actinin-1 (clone AT6.172) were from Santa Cruz Biotechnology and Millipore, respectively. The serum raised against a peptide common to both mouse ASB2 isoforms (1PLA) has been previously described^37^.

### Flow cytometry analysis and cell sorting

cDCs subsets were segregated based on the expression of CD11c, CD4, and CD8α. DC activation was analyzed based on CD86 expression in CD11c^+^ subset. For enrichment of spleen DCs, CD11c positive microbeads were used as recommended by the manufacturer (Miltenyi Biotech). For quantification of FLNa expression by FACS, cells were fixed after extracellular staining with PBS 2.7% PFA 20 min at 4 °C and permeabilized by adding PBS 0.5% Triton X100 10 min at 4 °C. Cells were then immunostained with anti-FLNa and brilliant violet 421-conjugated donkey anti-rabbit antibodies (Biolegend). Flow cytometry analysis was performed with a LSRII cytometer (BD Biosciences) and cell sorting on a FACS ARIA II cytometer (BD Biosciences). Analyses of flow cytometry data were done using Flowjo (Treestar).

### Immunofluorescence microscopy and immunohistochemistry

Immunofluorescence microscopy was performed and analyzed as described^15^,^17^. Approximatively 100 FACS-sorted CD11c^+^ cells were analyzed for FLNa expression in 10-50 randomly chosen fields. For immunohistochemistry, mouse spleens were snap-frozen, embedded in optimal cutting temperature (O.C.T.) compound (Cell Path) and stored at –80 °C. Five-μm frozen sections were then obtained and fixed in cold acetone. Sections were stained with biotin anti-CD11c for detection of DCs and with anti-FLNa. Biotin-conjugated antibodies were detected with streptavidin-iFluor550 (AATBioquest). Secondary antibodies used were Alexa Fluor 488 coupled to goat anti-rabbit. Nuclei were visualized with 4′,6-diaminidino-2-phenylindole (DAPI). Images were acquired using a Zeiss Axio Imager M2 using a X40/1.3 oil Ph3 or a X63/1.3 oil DIC Plan Apochromat objectives (Zeiss). Images were acquired and processed using AxioVision software and AxioCam MRm camera (Zeiss).

### Transwell migration

Uncoated transwells (5-μm pore filter; Corning) were placed in 96-well plates filled with 0.15 ml of DC culture medium (IMDM, 1% L-glutamine, 1% penicillin-streptomycin, 0.2% bovine serum albumin, 0.1% β-mercaptoethanol) containing or not 100 ng/ml mouse SDF1α (Immunotools). 2.5 × 10^5^ enriched CD11c^+^ cells in DC culture medium were placed in the upper chamber of the transwell and incubated at 37 °C for 2 h. Cells that had migrated through the filter were recovered and stained with CD11c APC, CD4 FITC and CD8α PE antibodies. The percentages of each DC subsets of migrated cells and of input cells were assessed by flow cytometry after gating on the CD11c^hi^ population.

### Quantitative RT-PCR

RNA isolation, cDNA synthesis and real-time PCR with the Power SYBR Green mix were carried out as described^9^. Gene expression is presented as relative amount of mRNA normalized to Arbp.

### Protein Sample Processing

For western blot, whole cell extracts from HeLa cells that were mock-transfected or transfected with a mouse ASB2α vector using the JetPEI reagent (Polyplus transfection) were used as controls. Cytoplasmic BMDC extracts were carried out using the ProteoExtract Subcellular Proteome Extraction kit (Calbiochem)^13^. For MS analysis, protein extracts from BMDCs were reduced in Laemmli buffer (final concentration of DTT at 30 mM) for 30 min at 56 °C, and cysteine residues were alkylated by addition of iodoacetamide at a final concentration of 90 mM for 30 min at room temperature in the dark. Then 50 μg of samples were loaded on one-dimensional SDS-PAGE gels (separating gel: 10% acrylamide; stacking gel: 4.4% acrylamide). No fractionation was performed, and the electrophoretic migration was stopped as soon as the sample entered the separating gel. The gels were briefly stained using Coomassie Blue (Instant Blue, Expedeon), and each single band, which contained the whole of each sample, was cut. In-gel digestion was performed as described^13^ and the dried tryptic peptides were dissolved in 2% acetonitrile, 0.05% trifluoroacetic acid (TFA).

### NanoLC-MS/MS Analysis

Peptides were analyzed by nanoLC-MS/MS using an Ultimate 3000 NRS system (Dionex) coupled to an LTQ-Orbitrap Velos mass spectrometer (Thermo Fisher Scientific). Five μl of sample were loaded on a C-18 precolumn (Dionex) at 20 μl/min in 5% acetonitrile/0.05% TFA. After desalting, the precolumn was switched online with the analytical C-18 column (in-house made C18 microcolumn, 75 μm ID × 50 cm packed with Reprosil-Pur C18-AQ 3 μm resin, Dr Maisch GmbH) equilibrated in 95% solvent A (5% acetonitrile, 0.2% formic acid) and 5% solvent B (80% acetonitrile, 0.2% formic acid). The peptides were eluted using a 5 to 50% gradient of solvent B during 300 min at 300 nl/min flow rate. The LTQ-Orbitrap Velos was operated in data-dependent acquisition mode with the XCalibur software. The 20 most intense ions per survey scan were selected for CID fragmentation and the resulting fragments were analyzed in the linear trap (LTQ). Dynamic exclusion was employed within 60 s to prevent repetitive selection of the same peptide.

### Database Search and Validation

The Mascot software v2.3.2 (Matrix Science) was used to perform database searches, in the case of later quantitative analysis by MFPaQ software. A peaklist was created for each sample and individual Mascot searches were performed. The data were searched against Mouse entries UniprotKB/SwissProt (version January 2014) protein database. Carbamidomethylation of cysteines was set as a fixed modification and oxidation of methionine was set as variable modification. Specificity of trypsin digestion was set for cleavage after Lys or Arg, and two missed trypsin cleavage sites were allowed. The mass tolerance in MS and MS/MS were set to 5 ppm and 0.6 Da, respectively, and the instrument setting was specified as “ESI-Trap”. False discovery rates (FDR) less than 5% for peptide identifications, and FDR less than 1% for protein identifications were applied for further data validation using the in-house developed Prosper module. For quantitative analysis using MaxQuant v1.5.0 software, raw data files from the mass spectrometer were directly loaded in the software. The Andromeda module was used to perform database searches against Mouse entries in UniprotKB/SwissProt protein database. Similar parameters as we had used for Mascot searches were set, as well as FDR values for further data validation.

### Data Quantification

Quantification analysis was performed using the label-free module implemented in the MFPaQ v4.0.0 software^16^ and using the MaxQuant v1.4.0.8 software^25^, in parallel. MFPaQ uses the validated identification results and the extracted ion currents (XICs) of the identified peptide ions in the corresponding raw files, based on their experimentally measured retention time (RT) and monoisotopic m/z values. Quantification of peptide ions was performed based on extracted XIC areas values, after re-alignment of the different runs, using as a starting point the experimentally derived RT (based on the MS/MS event) or a predicted RT value if the peptide was not sequenced in a given run. Normalization was performed by the software based on the median of the ratios calculated from all these XIC areas between a given run and a reference run. To perform protein relative quantification in different samples, a protein abundance index (PAI) was calculated (defined as the average of XIC area values for at most three intense reference tryptic peptides per protein). The 3 peptides exhibiting the highest intensities across the different samples were selected as reference peptides, and these peptides were used to compute the PAI of the protein in the different samples. If only 1 or 2 peptides were identified and quantified in the case of low-abundant proteins, the PAI was calculated based on their XIC area values). In the quantification interface of the MaxQuant software, LFQ (label free quantitative) was set as a parameter for the label-free quantification options and razor+unique peptides were used for protein quantification. In order to perform nLC-MS/MS run alignments, the parameters setting was specified as “Match between runs”. The intensity values were normalized using the median of all values from each experiment. In order to avoid quantitative errors, we applied a filtering on the quantitative output from both software. Only proteins quantified by 2 or more peptides and with quantitative data in all experiments were selected for further analyses. The subsets of quantifiable proteins are shown in Supplementary Table 1.

### Statistical analysis

p-values were calculated with the Mann-Whitney t-test or the unpaired t-test with Welch’s correction, as indicated in the figure legends.

## Additional Information

**How to cite this article**: Spinner, C. A. *et al.* Substrates of the ASB2α E3 ubiquitin ligase in dendritic cells. *Sci. Rep.* **5**, 16269; doi: 10.1038/srep16269 (2015).

## Supplementary Material

Supplementary Information

Supplementary dataset

## References

[b1] Fogg D. K. *et al.* A clonogenic bone marrow progenitor specific for macrophages and dendritic cells. Science 311, 83–87 (2006). 16322423 10.1126/science.1117729

[b2] Naik S. H. *et al.* Development of plasmacytoid and conventional dendritic cell subtypes from single precursor cells derived *in vitro* and *in vivo*. Nature Immunology 8, 1217–1226 (2007). 17922015 10.1038/ni1522

[b3] Onai N. *et al.* Identification of clonogenic common Flt3+M-CSFR+ plasmacytoid and conventional dendritic cell progenitors in mouse bone marrow. Nature Immunology 8, 1207–1216 (2007). 17922016 10.1038/ni1518

[b4] Waskow C. *et al.* The receptor tyrosine kinase Flt3 is required for dendritic cell development in peripheral lymphoid tissues. Nature Immunology 9, 676–683 (2008). 18469816 10.1038/ni.1615 PMC2746085

[b5] Naik S. H. *et al.* Intrasplenic steady-state dendritic cell precursors that are distinct from monocytes. Nature Immunology 7, 663–671 (2006). 16680143 10.1038/ni1340

[b6] Dudziak D. *et al.* Differential antigen processing by dendritic cell subsets *in vivo*. Science 315, 107–111 (2007). 17204652 10.1126/science.1136080

[b7] den Haan J. M. & Bevan M. J. Constitutive versus activation-dependent cross-presentation of immune complexes by CD8(+) and CD8(−) dendritic cells *in vivo*. The Journal of Experimental Medicine 196, 817–827 (2002). 12235214 10.1084/jem.20020295 PMC2194052

[b8] den Haan J. M., Lehar S. M. & Bevan M. J. CD8(+) but not CD8(−) dendritic cells cross-prime cytotoxic T cells *in vivo*. The Journal of Experimental Medicine 192, 1685–1696 (2000). 11120766 10.1084/jem.192.12.1685 PMC2213493

[b9] Lamsoul I. *et al.* ASB2alpha regulates migration of immature dendritic cells. Blood 122, 533–541 (2013). 23632887 10.1182/blood-2012-11-466649

[b10] Skaar J. R., Pagan J. K. & Pagano M. SCF ubiquitin ligase-targeted therapies. Nature reviews. Drug discovery 13, 889–903 (2014). 25394868 10.1038/nrd4432 PMC4410837

[b11] Lamsoul I., Uttenweiler-Joseph S., Moog-Lutz C. & Lutz P. G. Cullin 5-RING E3 ubiquitin ligases, new therapeutic targets? *Biochimie*, 10.1016/j.biochi.2015.08.003 (2015). 26253693

[b12] Jin J., Ang X. L., Shirogane T. & Wade Harper J. Identification of substrates for F-box proteins. Methods Enzymol 399, 287–309 (2005). 16338364 10.1016/S0076-6879(05)99020-4

[b13] Burande C. F. *et al.* A label-free quantitative proteomics strategy to identify E3 ubiquitin ligase substrates targeted to proteasome degradation. Molecular & cellular proteomics: MCP 8, 1719–1727 (2009). 19376791 10.1074/mcp.M800410-MCP200 PMC2709196

[b14] Heuze M. L. *et al.* ASB2 is an Elongin BC-interacting protein that can assemble with Cullin 5 and Rbx1 to reconstitute an E3 ubiquitin ligase complex. The Journal of Biological Chemistry 280, 5468–5474 (2005). 15590664 10.1074/jbc.M413040200

[b15] Heuze M. L. *et al.* ASB2 targets filamins A and B to proteasomal degradation. Blood 112, 5130–5140 (2008). 18799729 10.1182/blood-2007-12-128744 PMC2597609

[b16] Baldassarre M. *et al.* Filamins regulate cell spreading and initiation of cell migration. PloS one 4, e7830 (2009). 19915675 10.1371/journal.pone.0007830 PMC2773003

[b17] Lamsoul I. *et al.* Functional and structural insights into ASB2α, a Novel Regulator of Integrin-dependent Adhesion of Hematopoietic Cells. The Journal of biological chemistry 286, 30571–30581 (2011). 21737450 10.1074/jbc.M111.220921 PMC3162417

[b18] Lamsoul I., Erard M., van der Ven P. F. & Lutz P. G. Filamins but Not Janus Kinases Are Substrates of the ASB2alpha Cullin-Ring E3 Ubiquitin Ligase in Hematopoietic Cells. PloS one 7, e43798 (2012). 22916308 10.1371/journal.pone.0043798 PMC3423375

[b19] Sakane A. *et al.* Junctional Rab13-binding protein (JRAB) regulates cell spreading via filamins. Genes to Cells: Devoted to Molecular & Cellular Mechanisms 18, 810–822 (2013). 23890175 10.1111/gtc.12078

[b20] Razinia Z., Baldassarre M., Cantelli G. & Calderwood D. A. ASB2alpha, an E3 ubiquitin ligase specificity subunit, regulates cell spreading and triggers proteasomal degradation of filamins by targeting the filamin calponin homology 1 domain. The Journal of biological chemistry 288, 32093–32105 (2013). 24052262 10.1074/jbc.M113.496604 PMC3814802

[b21] Robbins S. H. *et al.* Novel insights into the relationships between dendritic cell subsets in human and mouse revealed by genome-wide expression profiling. Genome biology 9, R17 (2008). 18218067 10.1186/gb-2008-9-1-r17 PMC2395256

[b22] Miller J. C. *et al.* Deciphering the transcriptional network of the dendritic cell lineage. Nature Immunology 13, 888–899 (2012). 22797772 10.1038/ni.2370 PMC3985403

[b23] Luber C. A. *et al.* Quantitative proteomics reveals subset-specific viral recognition in dendritic cells. Immunity 32, 279–289 (2010). 20171123 10.1016/j.immuni.2010.01.013

[b24] Nakamura F., Stossel T. P. & Hartwig J. H. The filamins: organizers of cell structure and function. Cell Adh Migr 5, 1–10 (2011). 21169733 10.4161/cam.5.2.14401 PMC3084982

[b25] Cox J. & Mann M. MaxQuant enables high peptide identification rates, individualized p.p.b.-range mass accuracies and proteome-wide protein quantification. Nature biotechnology 26, 1367–1372 (2008). 10.1038/nbt.1511 19029910

[b26] Bouyssie D. *et al.* Mascot file parsing and quantification (MFPaQ), a new software to parse, validate, and quantify proteomics data generated by ICAT and SILAC mass spectrometric analyses: application to the proteomics study of membrane proteins from primary human endothelial cells. Molecular & Cellular Proteomics: MCP 6, 1621–1637 (2007). 17533220 10.1074/mcp.T600069-MCP200

[b27] Gautier V. *et al.* Label-free quantification and shotgun analysis of complex proteomes by one-dimensional SDS-PAGE/NanoLC-MS: evaluation for the large scale analysis of inflammatory human endothelial cells. Molecular & Cellular Proteomics : MCP 11, 527–539 (2012). 22518033 10.1074/mcp.M111.015230 PMC3412980

[b28] Belz G. T. & Nutt S. L. Transcriptional programming of the dendritic cell network. Nature reviews. Immunology 12, 101–113 (2012). 10.1038/nri3149 22273772

[b29] Klebanoff C. A. *et al.* Retinoic acid controls the homeostasis of pre-cDC-derived splenic and intestinal dendritic cells. The Journal of Experimental Medicine 210, 1961–1976 (2013). 23999499 10.1084/jem.20122508 PMC3782040

[b30] Beijer M. R. *et al.* A crucial role for retinoic acid in the development of Notch-dependent murine splenic CD8- CD4- and CD4+ dendritic cells. European journal of immunology 43, 1608–1616 (2013). 23519987 10.1002/eji.201343325

[b31] Caton M. L., Smith-Raska M. R. & Reizis B. Notch-RBP-J signaling controls the homeostasis of CD8- dendritic cells in the spleen. The Journal of Experimental Medicine 204, 1653–1664 (2007). 17591855 10.1084/jem.20062648 PMC2118632

[b32] Sekine C. *et al.* Differential regulation of splenic CD8- dendritic cells and marginal zone B cells by Notch ligands. International Immunology 21, 295–301 (2009). 19181931 10.1093/intimm/dxn148

[b33] Lewis K. L. *et al.* Notch2 receptor signaling controls functional differentiation of dendritic cells in the spleen and intestine. Immunity 35, 780–791 (2011). 22018469 10.1016/j.immuni.2011.08.013 PMC3225703

[b34] Kohroki J. *et al.* ATRA-regulated Asb-2 gene induced in differentiation of HL-60 leukemia cells. FEBS Lett 505, 223–228 (2001). 11566180 10.1016/s0014-5793(01)02829-0

[b35] Guibal F. C. *et al.* ASB-2 inhibits growth and promotes commitment in myeloid leukemia cells. The Journal of Biological Chemistry 277, 218–224 (2002). 11682484 10.1074/jbc.M108476200

[b36] Nie L. *et al.* Notch-induced Asb2 expression promotes protein ubiquitination by forming non-canonical E3 ligase complexes. Cell Research 21, 754–769 (2011). 21119685 10.1038/cr.2010.165 PMC3085721

[b37] Bello N. F. *et al.* The E3 ubiquitin ligase specificity subunit ASB2beta is a novel regulator of muscle differentiation that targets filamin B to proteasomal degradation. Cell Death Differ 16, 921–932 (2009). 19300455 10.1038/cdd.2009.27 PMC2709956

