# Supplementary material for: Substrates of the ASB2α E3 ubiquitin ligase in dendritic cells
Source: Sci Rep. 2015 Nov 5;5:16269. doi: 10.1038/srep16269 (PMC4633680; doi:10.1038/srep16269)
Supplement: Supplementary Information [file srep16269-s1.pdf]

Substrates of the ASB2α E3 ubiquitin ligase in dendritic cells

Camille A. Spinner, Sandrine Uttenweiler-Joseph, Arnaud Metais, Alexandre Stella, Odile Burlet-Schiltz, Christel Moog-Lutz, Isabelle Lamsoul & Pierre G. Lutz

Legend to supplemental figure 1

Figure S1: Reproducibility of individual measurements amongst biological replicates of ASB2$\alpha^{+/+}$ or ASB2$\alpha^{-/-}$ BMDCs. Protein intensities of all ASB2$\alpha^{+/+}$ (A and C) or ASB2$\alpha^{-/-}$ (B and D) BMDCs measured using MaxQuant (A and B) or MFPaQ (C and D) label-free quantitation algorithms are plotted against each other. Correlation between measurements is determined by Pearson coefficient (R).

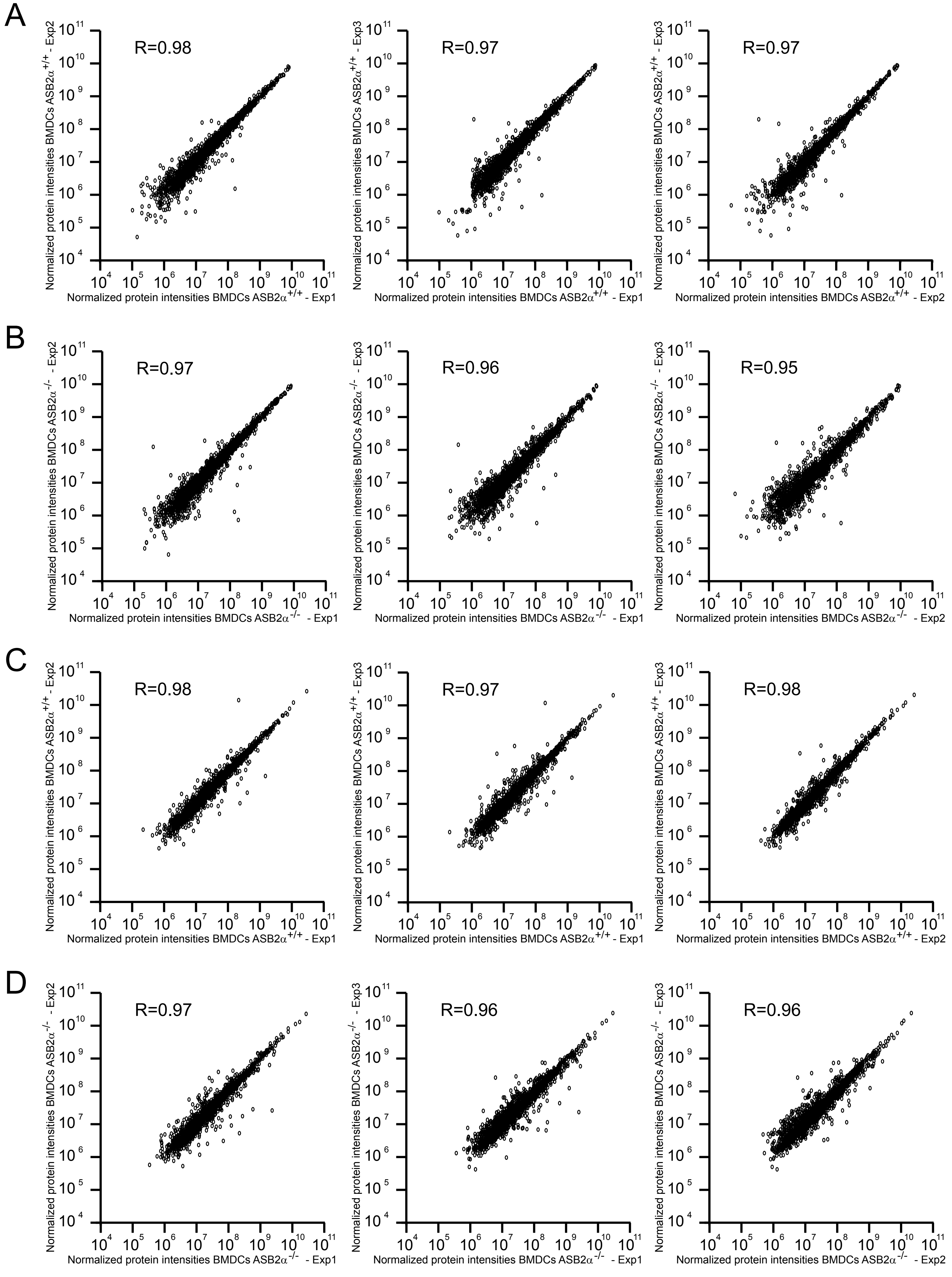
A
B
C
D
R=0.98
R=0.97
R=0.97
R=0.97
R=0.96
R=0.95
R=0.98
R=0.97
R=0.98
R=0.97
R=0.96
R=0.96
Normalized protein intensities BMDCs ASB2α+/+ - Exp1
Normalized protein intensities BMDCs ASB2α+/+ - Exp2
Normalized protein intensities BMDCs ASB2α+/+ - Exp3
Normalized protein intensities BMDCs ASB2α-/- - Exp1
Normalized protein intensities BMDCs ASB2α-/- - Exp2
Normalized protein intensities BMDCs ASB2α-/- - Exp3

Figure S1
